# Cathodic Exfoliation of Various Graphite Materials in Potassium Chloride Electrolyte

**DOI:** 10.3390/molecules30153151

**Published:** 2025-07-28

**Authors:** Md Habibullah Dalal, Nuwan Hegoda Arachchi, Chong-Yong Lee, Gordon G. Wallace

**Affiliations:** Intelligent Polymer Research Institute, AIIM Facility, Faculty of Engineering and Information Science, University of Wollongong, Wollongong, NSW 2500, Australia

**Keywords:** cathodic exfoliation, graphene, graphite sources, degree of graphitization

## Abstract

Cathodic exfoliation of graphite has emerged as an attractive method to synthesize high-quality and lo- defect graphene. Here, it is demonstrated that the type of starting graphite material influences the properties of exfoliated graphene. Graphite foil, natural graphite, and graphite rods were examined in the exfoliation processes performed in 3.0 M KCl at −15 V. The use of a graphite foil facilitates the rapid cathodic exfoliation process in comparison with structurally more compact natural graphite and graphite rods. For the graphite foil, the cathodically exfoliated graphene exhibits a low defect density (*I*_D_/*I*_G_ of 0.09, a C/O ratio of 35) with graphite exfoliation yield of 92.8%. In contrast, the exfoliated graphene from natural graphite exhibits an *I*_D_/*I*_G_ of 0.15, a C/O ratio of 28, and a graphite exfoliation yield of 30.5%, whereas graphene from graphite rod exhibits an *I*_D_/*I*_G_ of 0.86, a C/O ratio of 30, and a graphite exfoliation yield of 19.5%. The dense structure of natural graphite and graphite rods led to longer exfoliation times. Exfoliation of graphite rods produced few-layer graphene with the smallest sheet size, whereas natural graphite and graphite foil yielded multilayer graphene with larger sheets. This study demonstrates the feasibility of using aqueous-based cathodic exfoliation to produce graphene from various graphite sources, leading to variations in sheet thickness, size, defect density, and solvent dispersibility.

## 1. Introduction

Since the demonstration of the mechanical exfoliation of 2D carbon allotrope graphene from a highly oriented pyrolytic graphite (HOPG) in 2004 by Novoselov and Geim [1], it has become one of the most intensively studied materials [2,3]. A wide range of graphene production methods have been developed; however, developing a large-scale production method remains a challenge [4]. Over the recent years, electrochemical exfoliation of graphite has emerged as a simple, low cost, and scalable method to produce graphene [5,6,7,8,9,10,11]. The electrochemical exfoliation approach produces graphene with variations in levels of oxygen content, edge defect, and electrical conductivity, which depend on parameters such as graphite sources, the polarity of applied potential, and the electrolytes [9,10,11].

The anodic exfoliation of graphite in aqueous electrolytes has been studied extensively in recent years and is a considerably more developed method [11,12,13,14]. On the contrary, the cathodic exfoliation of graphite until recently has been limited to non-aqueous electrolyte in producing low-defect graphene through a reductive process [15,16,17]. Our studies show the feasibility of performing cathodic exfoliation of graphite in aqueous media using salts as simple as KCl and K_2_SO_4_ to produce low-defect graphene using graphite foils [18,19]. Electrochemical anodic exfoliation of graphite has been applied to various types of graphite, such as highly oriented pyrolytic graphite (HOPG), graphite foil, graphite rods, and powdered graphite [11,20]. It was found that the shape, size, and crystallinity of raw graphite materials play an essential role in controlling the quality of the anodically exfoliated graphene materials [11].

HOPG is an artificial graphite that possesses high crystallinity. The electrochemical exfoliation of HOPG using a wide range of electrolytes such as sulfuric acid and its salts [21,22,23,24], imidazolium and related ionic liquids [25,26,27], and perchlorate [15] were studied to produce graphene, where the produced graphene was mostly oxygen-functionalized [15,21,22,24,25,26]. However, because of its high cost, HOPG is only suitable and commonly employed for fundamental research or small-scale production. On the other hand, graphite rods are the most widely used form of graphite. The rods are available in diameters between 0.5 and 10 cm, and with variety of heights [11]. The electrochemical exfoliation of graphite rods using various electrolytes such as sulfuric acid and its salts [12,28] has been studied to produce anodically exfoliated graphene [17,28,29,30,31].

As a flexible sheet, graphite foil commonly has a thickness of ~0.5–2.0 mm [11,32]. Graphite foil can be produced either by pressing the expanded graphite or by carbonization and graphitization of polyimide foil. The latter exhibits superiority in electrical and thermal conductivity due to its higher crystallinity as well as suitability for electrochemical treatment [11]. Graphite foil is one of the most commonly used graphite sources for anodic exfoliation to produce graphene in sulfuric acid or sulphate-containing electrolytes [12,33,34]. A two-step exfoliation procedure was also employed, whereby the first step was achieving electrochemical graphite intercalation compound (GIC) formation in concentrated sulfuric acid, and the second step was exfoliating the GICs in ammonium sulphate [35] or in 50% sulfuric acid [36].

Natural graphite can be found in the form of natural rock or in crushed form as graphite powder during and after purification processes [11]. Palletized graphite powder was used in anodic exfoliation, but exfoliated graphene was typically non-uniform [12]. Improved performance was achieved by designing an electrode to function under a confined space to allow efficient and uniform intercalation and exfoliation [37,38]. For example, exfoliation of natural graphite powder was performed with graphite flakes wrapped in metal mesh [38,39], in a bag or container [40,41,42], in a dialysis membrane bag [41], or in a mesh filter bag [43].

Considering the impact of the source of graphite materials on anodic exfoliation, it would be of interest to examine such effects in cathodic exfoliations to produce graphene [18]. The advantage of the cathodic exfoliation process, compared to its anodic counterpart, lies in its reductive nature, which produces graphene with fewer defects and higher conductivity. Additionally, aqueous-based cathodic exfoliation offers faster exfoliation rates and uses environmentally friendly electrolytes, making it suitable for large-scale production. In this study, electrochemical cathodic exfoliation was performed to produce graphene in aqueous electrolytes from three different types of graphite: foil, rod, and natural graphite, which differ in their physical and chemical properties. The impact of the source of graphite materials on the resultant properties of exfoliated graphene such as its lateral size, processability in solvent systems, as well as other physical and thermal properties was investigated.

## 2. Results and Discussion

The influence of the graphite starting materials on the properties and quality of the electrochemically cathodic-exfoliated graphene has been examined. A cathodic potential of 15 V was applied to a graphite cathode in a 3.0 M KCl aqueous electrolyte. Our recent study on the successful cathodic exfoliation of a graphite foil employed −10.0 V in 2.0 M KCl electrolyte [18], but under these conditions it was found that the graphite rod and natural graphite could not be effectively exfoliated. However, increasing the voltage and KCl concentration to −15.0 V and 3.0 M KCl, respectively, enabled exfoliation of the graphite rod and natural graphite. To consistently compare all the exfoliated graphene samples, these new parameters were applied to all three types of graphite electrodes.

Graphite foil possesses a large number of nanometer-sized interlayer voids and packing imperfections such as localized expanded interlayer spacing areas, which arise from its production process. There are visible flaws such as folds, overlaps, or wrinkles appearing on the electrode surfaces [20]. In contrast, graphite rods and natural graphite (rock, powder, or flakes) consist of graphene layers that are very tightly stacked onto each other, leaving essentially no interlayer voids or openings [19,20]. We have previously proposed the mechanism of aqueous cathodic exfoliation, which depends on the sizes of cations and the applied potential as driving forces in generating hydrogen [18]. Hydrated cations may play a key role in being intercalated into the graphene layers of graphite foil. Here, similar positively charged graphene layers in graphite results in a repulsive force, which open up the edges or grain boundaries, thus expanding the graphite. As the process is an electrochemical reductive reaction in an aqueous electrolyte, proton reduction occurs, which generates hydrogen. The gas produced in a large quantity between the interlayer spaces would then assist in the expansion and subsequent exfoliation of the graphite. This would suggest that to overcome the compactness of graphite rods and natural graphite, a larger cathodic potential and a higher KCl concentration are required to accelerate the intercalation and gas-bubble-assisted exfoliation processes.

Our previous report demonstrated that aqueous cathodic exfoliation can occur using electrolytes having quaternary ammonium-based cations of a certain intermediate size, for example, tetrapropylammonium or hexyltrimethylammonium, for graphite foil but not for graphite rods or natural graphite [19]. It was proposed that graphite foil has pre-expanded edges and interlayer voids that allow larger-sized quaternary ammonium-based cations to intercalate, and this structure is absent in graphite rods and natural graphite. This study demonstrates that it is possible to exfoliate graphene from graphite rods and natural graphite by applying a sufficiently high driving voltage of −15.0 V in 3.0 M KCl.

**Figure 1:**
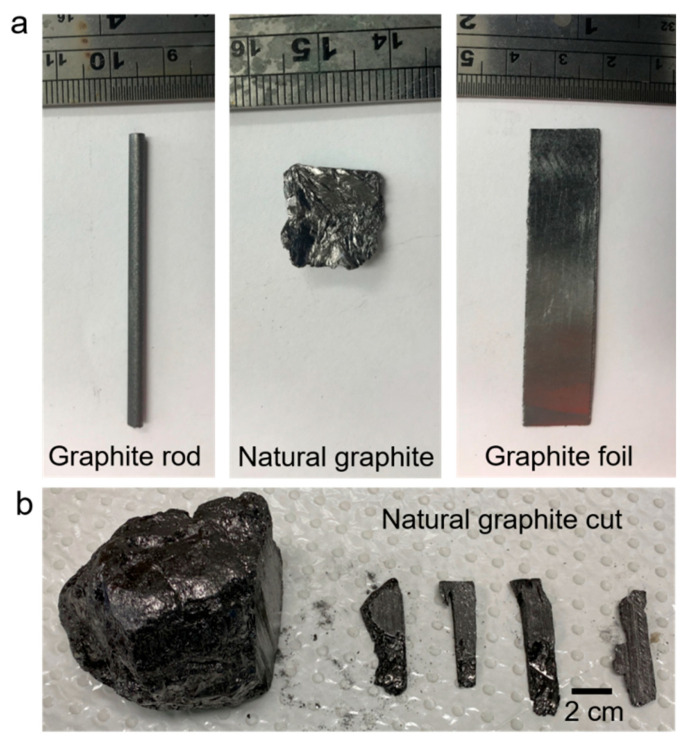
Optical photographs of graphite electrodes investigated in this study. (**a**) Graphite rod, natural graphite, and graphite foil; (**b**) natural graphite and the smaller cut pieces.

The properties of cathodically exfoliated graphene were examined and characterized using three different graphite sources, graphite foil, graphite rods, and natural graphite, as shown in Figure 1. Due to the irregular shape of natural graphite, accurately determining the immersed surface area for exfoliation posed a challenge. To address this, bulk natural graphite was shaped into an electrode-like form (Figure 1b) and immersed into the electrolyte to expose a surface area of 1 cm^2^, ensuring consistency with cathodic exfoliation studies using graphite rod and foil samples.

The graphite exfoliation yield was calculated as the mass ratio of exfoliated graphite to the mass of the graphite starting material, as reported previously [18]. The graphite foil shows a high graphite exfoliation yield of 92.8%, whereas natural graphite and graphite rods have lower graphite exfoliation yields of 30.5% and 19.5%, respectively. The exfoliation rates for the graphite foil, natural graphite, and graphite rod were 0.73 g h^−1^, 0.08 g h^−1^, and 0.04 g h^−1^, respectively. A comparison of the yield, production rate, oxygen content, and defect density of graphene prepared from the various graphite sources such as graphite foil, natural graphite (graphite flakes), graphite rod, and HOPG by various electrochemical exfoliation methods is presented in Supplementary Table S1.

**Figure 2:**
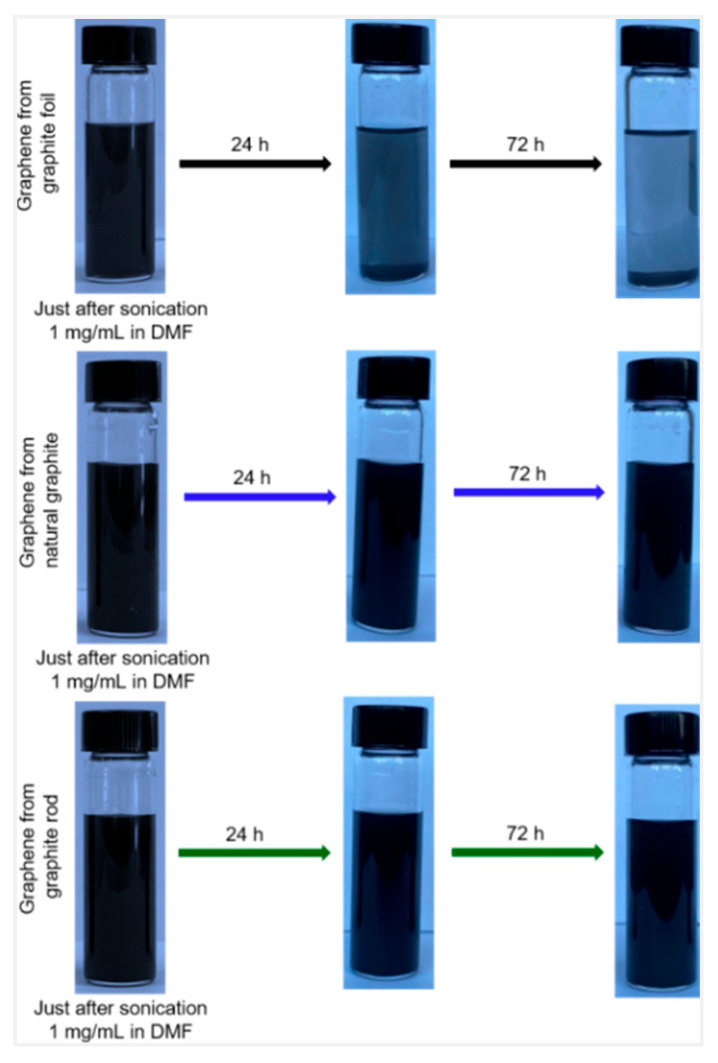
Photograph of dispersed cathodically exfoliated graphene from graphite foil (**top**), graphene from graphite rod (**middle**), and cathodic graphene from natural graphite (**bottom**) in DMF solution (concentration ~1 mg/mL). From left to right: immediately just after sonication, after 24 h, and after 72 h.

All three electrochemically characterized graphenes were dispersed in DMF using ultrasonication for 2 h, and the dispersions were maintained undisturbed for 24 h. The top portions of all the three graphenes dispersed in DMF were used for further characterization. Graphene that was prepared from a graphite rod and natural graphite (~1 mg/mL) after ultrasonication remained stable for at least 3 days without any obvious agglomeration or settling. In contrast, graphene synthesized from graphite foil was not stable and agglomerated within 24 h (Figure 2). The agglomeration is greater after 72 h. This is consistent with previously reported results, where cathodically exfoliated graphene using a graphite foil in potassium chloride electrolyte has poor dispersibility [18,19].

### 2.1. Atomic Force Microscopy Analysis

**Figure 3:**
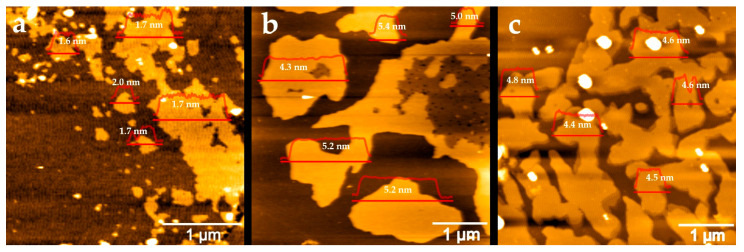
AFM images of the graphene samples obtained from graphite rod (**a**), natural graphite (**b**), and graphite foil (**c**). Line profiles within the AFM images represent the cross-sectional thickness (Z-height) of the graphene samples. The average thickness across the corresponding areas is shown next to the line profiles.

The thickness and lateral dimensions of the graphene sheets were characterized using atomic force microscopy (AFM), as shown in Figure 3. Graphene samples were drop-cast onto silicon wafer surfaces for AFM characterization. Graphene exfoliated from graphite rods exhibited a wide range of sheet sizes, from 0.1 to 1.5 µm, with an average thickness of 1.7 ± 0.2 nm. Given that a single graphene layer has a thickness of approximately 0.345 nm, this corresponds to few-layer graphene (~5 layers). In contrast, graphene obtained from natural graphite had an average thickness of 5.0 ± 0.4 nm and larger sheet sizes ranging from 1 to 3 µm. Graphene from graphite foil showed an average thickness of 4.6 ± 0.2 nm, with irregularly shaped sheets measuring 1 to 2 µm. The thicknesses of graphene from both natural graphite and graphite foil exceed 10 layers, commonly identified as multilayer graphene or graphene nanoplatelet.

### 2.2. X-Ray Diffraction Analysis

**Figure 4:**
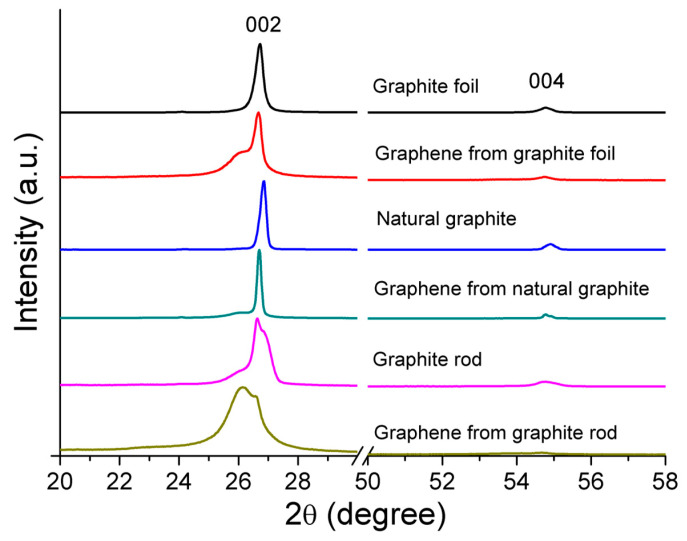
XRD patterns of the raw graphite foil, natural graphite, and graphite rod, normalized to their peak intensity at (002), and their corresponding electrochemical cathodic-exfoliated graphenes.

XRD measurement was used to characterize the crystalline nature and phase purity of the raw graphite and their respective cathodic-exfoliated graphene materials. Figure 4 shows the XRD patterns of the graphite foil, natural graphite, and graphite rod, which exhibit strong peaks at 26.72°, 26.85°, and 26.65°, respectively, and correspond to the (002) and other peaks with relatively weak intensity at 54.8°, 54.9°, and 54.7°, respectively, which correspond to the (004) reflection planes of graphite. The characteristic XRD peaks for the cathodic-exfoliated graphenes for the (002) reflection planes varied with the source of graphite. A high degree of graphitization is indicated by sharp XRD peaks, reflecting the superior crystalline structure of the graphite and its enhanced diffraction compared to samples with broader peaks [44]. The graphite foil and graphite rod display sharp peaks, indicating high crystallinity, whereas the broader peak observed in the graphite rod reflects a lower degree of graphitization. Upon exfoliation, graphene derived from graphite foil exhibits a slight left shift, along with the emergence of a left shoulder peak. Graphene obtained from exfoliated natural graphite shows enhanced peak sharpness and a subtle left shoulder peak. In contrast, graphene from the graphite rod displays the most pronounced left shift and the broadest peak, indicating a more amorphous nature. Another characteristic peak (004) is less intense compared to the peak at (002). The enlarged view of these peaks (see Figure S1) shows that all exfoliated graphene samples exhibit slightly left-shifted and less-intense peaks compared to their corresponding graphite sources.

### 2.3. Raman Spectra Analysis

**Figure 5:**
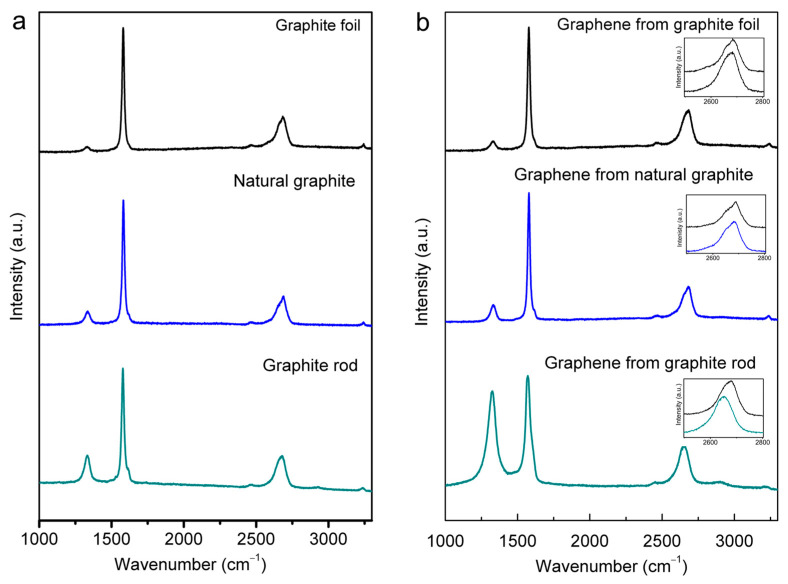
Raman spectra (excited by 633 nm laser) of (**a**) graphite rod, natural graphite, and graphite foil, and (**b**) graphene samples obtained from graphite rod, natural graphite, and graphite foil, In the insets, magnified 2D band range for corresponding cathodic-exfoliated graphene (lower trace) and the respective source graphite (upper trace).

Figure 5 presents the Raman spectra of the starting graphite materials and the cathodically exfoliated graphene from these raw graphites that were obtained using a 633 nm excitation laser, which show the structural quality of the respective materials. All spectra display typical Raman D, G, and 2D bands at around 1330, 1580, and 2670 cm^−1^, respectively [20,45,46]. The G peak refers to the first-order scattering of the E_2g_ mode of sp^2^ carbon atoms. The prominent D peak originates from the breathing mode of the sp^2^ carbon atoms, which is activated by the existence defects, including edges and structural disorders [47].

The *I*_D_/*I*_G_ ratios are widely adopted as a quantitative measure of the level of defects present in the graphitic structures [46,48]. The precursor graphites had increasing structural quality in the following order: graphite rod < natural graphite < graphite foil. The *I*_D_/*I*_G_ ratios were 0.24, 0.13, and 0.05 for the graphite rod, natural graphite, and graphite foil, respectively (Figure 5a). The calculated *I*_D_/*I*_G_ values of the different cathodic-exfoliated graphene samples are 0.86, 0.15, and 0.09 for graphite rod, natural graphite, and graphite foil, respectively, and are provided in Table S2. A noticeable difference is a significantly higher *I*_D_/*I*_G_ ratio for exfoliated graphene obtained from the graphite rod. This indicates this graphene had significantly higher edge defects, which is consistent with better dispersibility in DMF solvent. This may also be attributed to the smaller graphene sheet size obtained from this graphite source (see Figure S1).

The Raman 2D band is located at about 2700 cm^−1^. The shape of the 2D band for graphites in bulk, non-exfoliated form is markedly asymmetrical (see Figure 5b, upper trace in each of the insets), and this is consistent with previously reported data [45]. As is consistent with the D and G bands, the 2D band also shifted more prominently for graphene exfoliated from the graphite rod relative to those exfoliated from natural graphite and graphite foil (Figure 5b, lower trace in the inset plots). Such features are consistent with these samples comprising exfoliated graphene sheets [46,48,49] and are in agreement with the results obtained by XRD (Figure 4).

### 2.4. Thermogravimetric Analysis

**Figure 6:**
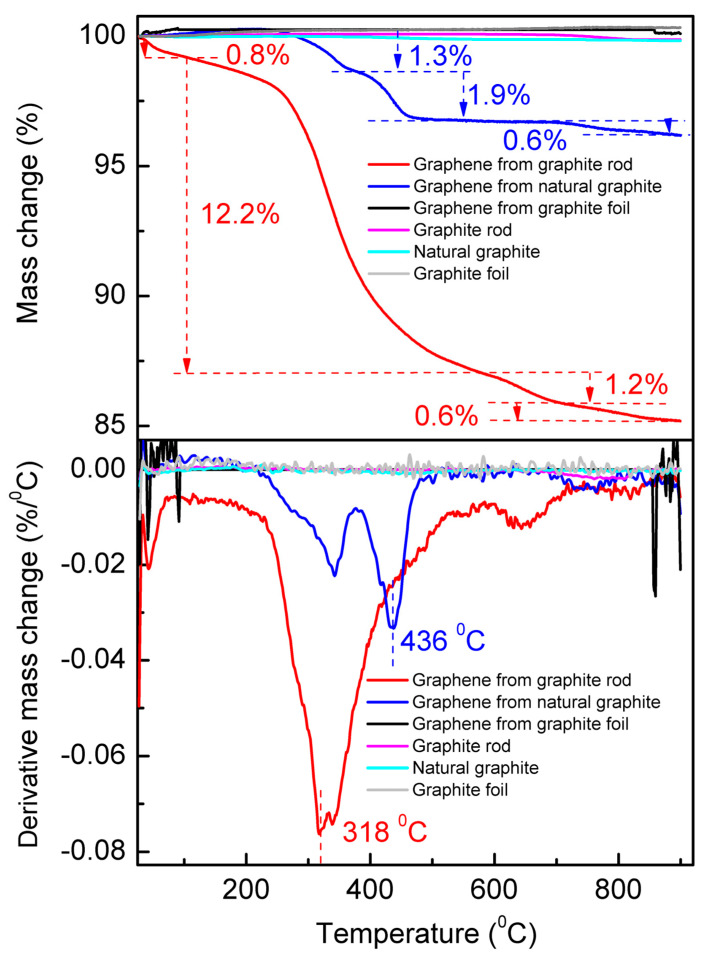
TGA (**top**) and DTG (**bottom**) measurements of graphite rod, natural graphite, graphite foil, and the cathodically exfoliated graphene from these raw graphites from room temperature (25 °C) to 900 °C under N_2_ atmosphere.

The TGA measurements were performed from room temperature (25 °C) to 900 °C under N_2_ atmosphere at a heating rate of 10 °C min^−1^ (Figure 6). The top panel shows the TGA curve, and the bottom panel is their corresponding first-order derivative thermogravimetric (DTG) curve that presents the mass loss percentage and the temperature of maximum mass change rate (T_m_). The DTG curves show a general trend with T_m_ increasing to higher temperatures as the average particle size increases [50]. In our case, the T_m_ of the cathodic-exfoliated graphene from the graphite rod is 318 °C, whereas the T_m_ of the cathodic-exfoliated graphene from natural graphite is 436 °C. The graphite rod, natural graphite, and graphite foil remained stable in a nitrogen atmosphere during heating, as graphite is known for its high thermal stability and resistance to decomposition (see Figure S2) [51,52]. Cathodically exfoliated graphene from the graphite foil also shows no significant changes during the heating process, likely due to its lower defect density [18]. The DTG curves revealed four and three distinct mass loss events during the thermal degradation of cathodically exfoliated graphene derived from the graphite rod and natural graphite, respectively, as shown in Figure 5.

The mass loss observed in the cathodically exfoliated graphene from the graphite rod can be attributed to the decomposition of its components at specific temperatures. The first weight loss occurs at ≤100 °C due to desorption of water and residual solvents, which accounts for around 0.8% of the weight loss [53]. The second mass loss event occurs within the range of 142–566 °C with a mass loss of 12.2%, and the T_m_ is within this temperature range. This major weight loss is attributed to decomposition of weakly bound oxygen functional groups (e.g., hydroxyl, carboxyl) and more stable oxygen groups (e.g., epoxy, carbonyl). The third mass loss of 1.8% is in the temperature range of 582–720 °C, indicating possible breakdown of defective carbon structure, and the fourth mass loss occurs at 750–900 °C of 0.6%, possibly due to the decomposition of the most intact graphene framework.

Compared to cathodically exfoliated graphene derived from a graphite rod, which exhibits a total weight loss of approximately 15%, graphene obtained from natural graphite shows a significantly lower weight loss of around 4%. The first noticeable weight loss event occurs within the temperature range of 215–378 °C, accounting for 1.3% of the mass loss in the natural-graphite-derived graphene. The second and third weight loss events occurred at 376–511 °C and 686–900 °C, accounting for 1.9% and 0.6% of the mass loss, respectively. These weight loss events indicate relatively smaller amounts of oxygen functional groups for this graphene compared to graphite-rod-derived graphene.

Consistent with findings from other characterization techniques, cathodically exfoliated graphene from graphite foil exhibits a very low defect density and a high carbon-to-oxygen (C/O) ratio. Consequently, it undergoes only negligible weight loss during thermal decomposition.

### 2.5. X-Ray Photoelectron Spectroscopy

**Figure 7:**
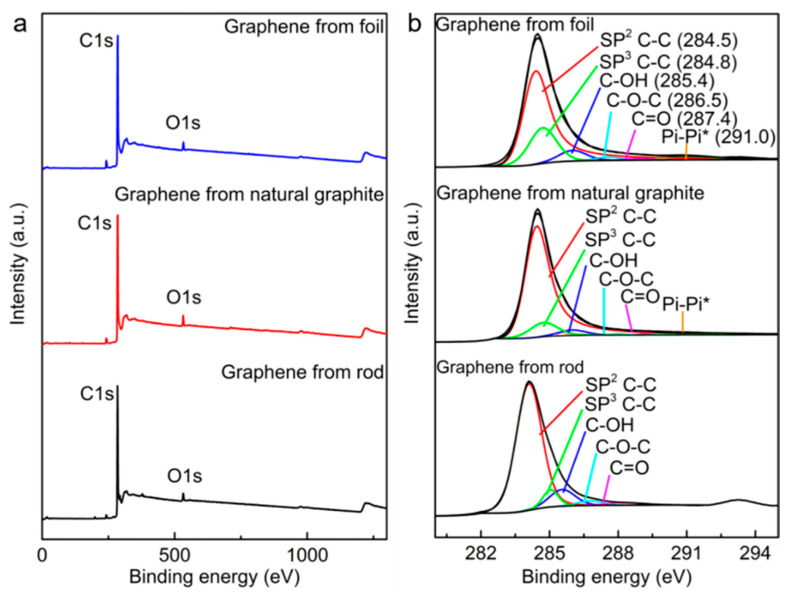
(**a**) XPS survey and (**b**) high-resolution C 1s spectrum of cathodically exfoliated graphene from graphite rod, natural graphite, and graphite foil.

X-ray photoelectron spectroscopy (XPS) was performed to investigate the chemical composition of the cathodically exfoliated graphene, as shown in Figure 7. The deconvolution of the C 1s spectra (Figure 7b) reveals peaks at 284.5 eV and 284.8 eV, associated with graphitic sp^2^ C–C and sp^3^ C–C, respectively, and are present for all graphenes. This occurs along with the π–π* shakeup peak at approximately 291 eV. Peaks attributed to oxygen-containing groups such as C–OH, C–O–C, and C=O are relatively weaker in all cathodically exfoliated graphenes [18,19]. The C/O atomic ratios of cathodically exfoliated graphene from the graphite rod, natural graphite, and graphite foil were 30, 28, and 35, respectively (Table 1). The XPS analyzed data are summarized and provided in Table S2. The survey scan of the source graphites and the C/O ratio of these raw graphite materials are provided in Figure S3 and Table S3, respectively. The XPS data are consistent with the Raman, TGA, and dispersibility analyses of the graphene exfoliated from graphite foil. This sample exhibited the lowest defect density, with an *I*_D_/*I*_G_ of 0.09, and the highest C/O ratio of 35, indicating minimal oxidation and surface defects. In contrast, graphene derived from the graphite rod showed the highest defect density (*I*_D_/*I*_G_ of 0.86) while still maintaining a relatively moderate C/O ratio of 30. This higher defect level can be attributed to the smaller graphene sheet size, which increases the proportion of edge sites, thereby amplifying the apparent defect density. This interpretation is further supported by the dispersion stability of graphene from the graphite rod, which is likely enhanced by both the smaller lateral dimensions of the sheets and the abundance of reactive edge sites.

## 3. Experimental Section

### 3.1. Materials

Three different types of graphite were employed, namely, graphite foil, graphite rod, and natural graphite. Graphite foil (0.254 mm, 99.8 % metal basis) was purchased from Alfa Aesar (Ward Hill, MA, USA). Graphite rod (diameter 3 mm, 99.995% trace metal basis) was purchased from Sigma-Aldrich (St. Louis, MO, USA). Natural graphite was obtained from Sri Lankan Vein Graphite (Vancouver, BC, Canada). Analytical-grade chemicals, KCl (Chem Supply) and dimethylformamide (DMF) solvent (Sigma Aldrich) were used as-received without further purification. Milli-Q (resistivity of 18.2 MΩ.cm at 25 °C) water was used in all aqueous-based experiments.

### 3.2. Cathodic Exfoliation Experiments

The following dimensions of graphite (Figure 1) were employed in cathodic exfoliation experiments: graphite foil (1.0 cm × 1.0 cm × 0.0254 cm), graphite rod (diameter: 0.3 cm; length: 40 mm), and natural graphite (approximately 0.5 cm × 1.8 cm × 0.6 cm). All three graphite materials had an exposed surface area of 1 cm^2^ when immersed in the electrolyte. Electrochemical exfoliation was conducted in a two-electrode system. Graphite foil, rod, or natural graphite and a Pt foil were immersed in an aqueous 3.0 M KCl solution, which were used as the cathode and anode, respectively. The Pt foil was placed parallel to the graphite sample at a distance of about 2 cm. Then, a cathodic voltage (typically −15.0 V) was applied to the graphite electrode using a TENMA 72-2550 programmable DC power supply (TENMA, Tokyo, Japan). Upon completion of electrochemical exfoliation, the products were filtered through filter papers, washed several times with Milli-Q water via vacuum filtration, and dried overnight at room temperature. The resultant exfoliated graphene from the foil, rod and natural graphite was dispersed in DMF, each in a separate sample vial. The dispersions were maintained for 24 h, and the upper part of the dispersions was used for further characterization. Some samples were maintained for up to 72 h to examine the dispersion stability.

### 3.3. Material Characterization

The samples were characterized by atomic force microscopy (AFM), X-ray diffraction (XRD), Raman spectroscopy, field-emission scanning electron microscopy (FE-SEM), thermogravimetric analysis (TGA), and X-ray photoelectron spectroscopy (XPS). The samples were examined using an Asylum MFP-3D Origin AFM (Oxford Instruments, Oxford, UK) operating in AC mode with MikroMasch HQ:NSC15/Al BS cantilevers (spring constant ~ 40 N/m, MikroMasch, Wilsonville, OR, USA). AFM images analysis was performed using MFP-3D 18.18.33 software and Gwyddion 2.68 software. XRD (GBC MMA diffractometer) was carried out with Cu Kα radiation at a scan rate of 2 degree per minute. Raman spectroscopy was performed with a Horiba Jobin Yvon LabRam HR Evolution Raman Spectrometer using a 633 nm laser line (Horiba, Kyoto, Japan). The surface morphologies of graphene sheets were recorded using SEM (JEOL JSM-7500FA, JEOL, Tokyo, Japan). The TGA (TGA 209—NETZSCH, Selb, Germany) measurements were carried out at a heating rate of 10 °C min^−1^ ranging from room temperature to 900 °C under N_2_ atmosphere. XPS spectra were collected by illuminating the samples with a non-monochromatic X-ray source (Omnivac, Kaiserslautern, Germany) using Al Kα (1486.6 eV) radiation, and the photoemission was collected by a Nexsa XPS System (Thermo Fisher Scientific, Waltham, MA, USA). Survey scans were carried out with a pass energy of 200 eV, and a step size of 1 eV was used; region scans were performed using a pass energy of 50 eV with a step size of 0.1 eV. Each spectrum was acquired by performing about 20 to 30 repeated scans. XPS spectra were calibrated by referencing the primary C 1s peak to 284.5 eV in accordance with the literature.

## 4. Conclusions

This work demonstrates that aqueous-based cathodic exfoliation is a simple and versatile method for producing graphene from various graphite sources. Under optimized conditions (–15 V and 3.0 M electrolyte concentration), exfoliation of the structurally compact natural graphite and graphite rod is achievable, although it proceeds at a slower rate and with lower yield compared to graphite foil. The source of the graphite material significantly influenced the properties of the resulting graphene, as evidenced by the production of few-layer graphene from graphite rods and multilayer graphene or graphene nanoplatelets from natural graphite and graphite foil. The graphene from the exfoliated graphite rod had the highest defect density, smallest sheet size, and the highest dispersibility in DMF. This contrasts with the larger sheet sizes of the graphene produced from graphite foil and natural graphite, which have less edge defects and hence poor dispersibility. In conclusion, the aqueous-based cathodic exfoliation approach is applicable to a wide range of graphite materials. The characteristics of the starting graphite not only influence the rate and yield of graphene production but also affect key properties of the resulting graphene, including layer thickness, defect density, sheet size, and dispersibility in solvents.

## Figures and Tables

**Table 1:** C/O ratio of aqueous cathodic-exfoliated graphene from graphite rod, natural graphite, and graphite foil.

Sample	Element		C/O (Ratio)
	C1s (Atomic %)	O1s (Atomic %)	
Cathodic graphene from graphite rod	96.8	3.2	30
Cathodic graphene from natural graphite	96.5	3.5	28
Cathodic graphene from graphite foil	97.2	2.8	35

## Data Availability

The data presented in this study are available upon request from the corresponding author.

## Supplementary Materials

The following supporting information can be downloaded at: https://www.mdpi.com/article/10.3390/molecules30153151/s1. Figure S1. XRD patterns of the raw graphite foil, natural graphite, and graphite rod, normalized to their peak intensity at (004), and their corresponding electrochemical cathodic-exfoliated graphenes. Figure S2. The TGA (top) and DTG (bottom) measurements of graphite rod, natural graphite, and graphite foil from room temperature (25 °C) to 900 °C under N_2_ atmosphere. Figure S3. XPS survey scans of graphite rod, natural graphite, and graphite foil. Table S1. Comparison of the yield, production rate, oxygen content, and defect density of graphene prepared from various graphite sources by electrochemical exfoliation methods. Table S2. Raman characteristics of cathodically exfoliated graphenes obtained from different types of graphite: peak D, G, and 2D positions, their intensity ratio, and the calculated crystallite sizes. Table S3. C/O ratio of raw materials of graphite rod, natural graphite, and graphite foil. Refs [54,55,56,57,58,59,60,61,62,63,64,65,66,67,68,69,70] are cited in the Supplementary Materials.
