# Supplementary material for: Cathodic Exfoliation of Various Graphite Materials in Potassium Chloride Electrolyte
Source: Molecules. 2025 Jul 28;30(15):3151. doi: 10.3390/molecules30153151 (PMC12348432; doi:10.3390/molecules30153151)
Supplement: Supplementary file 1 [file molecules-30-03151-s001.zip › molecules-3736843-supplementary.pdf]

Supporting Information for

Cathodic Exfoliation of Various Graphite Materials in Potassium Chloride Electrolyte

Md Habibullah Dalal, Nuwan Hegoda Arachchi, Chong-Yong Lee * and Gordon G. Wallace *

Intelligent Polymer Research Institute, AIIM Facility, Faculty of Engineering and Information Science, University of Wollongong, Wollongong, NSW 2500, Australia; habib.buetchem@gmail.com (M.H.D.); nuwan@uow.edu.au (N.H.A.)

* Correspondence: cylee@uow.edu.au (C.-Y.L.); gwallace@uow.edu.au (G.G.W.)

Table S1 Comparison of the yield, production rate, oxygen content and defect density of graphene prepared from various graphite sources by electrochemical exfoliation methods.

Raw graphite	Electrolytes	Working potentials	Yield	Production rate (g h^{-1})	O content	I_D/I_G	Ref.
Graphite foil	1) 5 wt% H_2SO_4, 2) 0.1 M $(NH_4)_2SO_4$	+2.2V, 10 min +10 V	71%	~21	17.7%	1.48	[1]
Graphite foil	0.1M $(NH_4)_2SO_4$ solution	+10V, 10 min	75%	16.3	5.5%	0.25	[2]
Graphite foil	0.05 M oxone solution	+50 V, 4 min	60.1%	—	16.37%	1.24	[3]
Graphite foil	NaOH solution + p-phthalic acid	+10 V, 6-8 h	87.3%	—	22.6%	0.9	[4]
Graphite foil	1) 0.5 M Na_2SO_4, 2) 0.5 M H_2SO_4, 3) 0.5 M $LiClO_4$	+10 V, 20 min	—	—	10.2% 11% 20%	0.95 1.34 1.00	[5]
Graphite foil	0.1 M H_2SO_4	+10 V, 10 min	60 %	4.2	7.5%	0.4	[6]
Graphite foil	1) 1M NaOH 2) 0.5 M H_2SO_4	1) +10V, 10 h 2) +10 V, 1 h	56%	—	8.3%	0.29	[7]
Graphite foil	0.1 M $(NH_4)_2SO_4$ + 1 mg mL^{-1} TEMPO	+10 V, 10 min	75 %	15.1	3.8%	0.1	[8]
Graphite foil	1) 95 wt % H_2SO_4, 2) 0.1 M K_2SO_4	+10 V, 1 h	50%	—	16.7%	0.6	[9]
Graphite flakes	1 M H_2SO_4 + Saturated $(NH_4)_2SO_4$	0.6A, 24 h	39%	< 0.1	21%	~1	[10]
Graphite flakes	Dilute H_2SO_4 + melamine	±20 V, 10 min	25%	1.50	3.7%	0.45	[11]
Graphite flakes	1 M $LiPF_6$/PC	0 V, 4-8 h	80%	—	3.5%	0.45	[12]
Graphite flakes	1 M LiCl + Et_3NHCl/DMSO	CV: 0 V to -6 V	<30%	0.5-2	6.0%	0.3	[13]
Graphite flakes	1) H_2SO_4+HAc 2) 10 M H_2SO_4	+1V, 10 min +2V, 20 min	75%	—	3.9%	0.3	[14]
Graphite flakes	0.1 M $TBAClO_4$/PC	-20 V	91.5%	50	4.2%	0.05	[15]
Graphite rod	Ambient temperature molten salts	+3 V, 20 h	76%	<1 g/h	11.97%	0.60	[16]
Graphite rod	1 mM PSS solution	+ 5 V, 4 h	15%	< 1	—	0.65	[17]

Graphite rod	glycine + H_2SO_4 solution	+1 V, 5 min, +3 V, 5 min	—	—	11%	0.7	[18]
Graphite rod	0.1 M $TMABF_4$, $TEABF_4$, $TBABF_4$ in NMP	-5 V, 6000 s	<10%	—	7.96%	0.3	[19]
Graphite rod	melted LiCl at 800°C	-2.8 V, 30 min	70%	>14	4.4%	0.32	[20]
HOPG	0.1M $TBAPF_6$ / DMF	-20 V, 2 h	25%	—	4.6%	0.14	[21]
HOPG	$BMPTF_2N$ ionic liquid	-20 V, 10 h	25%	—	2.7%	0.05	[22]
HOPG	30 mg mL^{-1} LiClO4/PC	-15±5 V	70%	0.12	—	<0.1	[23]
Graphite foil	3.0 M KCl (aq) sol^n	-15 V, 5 min	93%	0.73	2.8%	0.09	**This work**
Natural Graphite		-15 V, 30 min	31%	0.08	3.5%	0.15	
Graphite rod		-15 V, 2 h	20%	0.04	3.2%	0.86	

Table S2 Raman characteristics of cathodically exfoliated graphenes obtained from different types of graphite: Peak D, G and 2D positions, their intensity ratio, and the calculated crystallite sizes.

Graphite/graphene material	ω_D (cm^{-1})	ω_G (cm^{-1})	ω_{2D} (cm^{-1})	I_D/I_G (Raman)
Graphite rod	1333	1579	2679	0.24
Graphene from graphite rod	1325	1571	2652	0.86
Natural graphite	1334	1582	2687	0.13
Graphene from natural graphite	1333	1578	2682	0.15
Graphite foil	1330	1581	2684	0.05
Graphene from graphite foil	1328	1578	2681	0.09

Table S3 C/O ratio of raw materials of graphite rod, natural graphite and graphite foil

Sample	Element		C/O (ratio)
	C1s (Atomic %)	O1s (Atomic %)	
Graphite rod	98.9	1.1	89.9
Natural graphite	95.9	4.1	23.4
Graphite foil	99.9	0.1	999

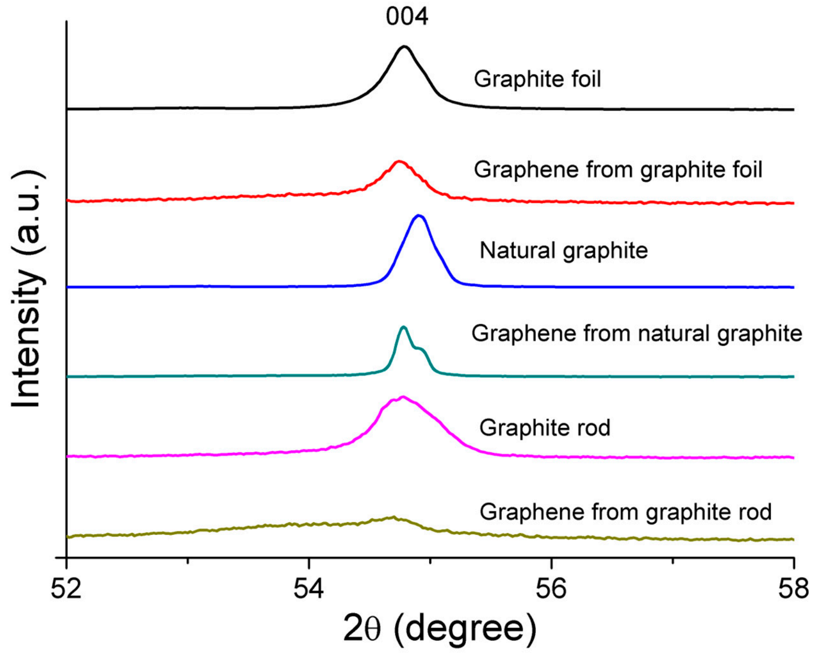

Figure S1. The enlarged of XRD patterns of the raw graphite foil, natural graphite and graphite rod, and their corresponding electrochemical cathodic exfoliated graphene shown in Figure 4.

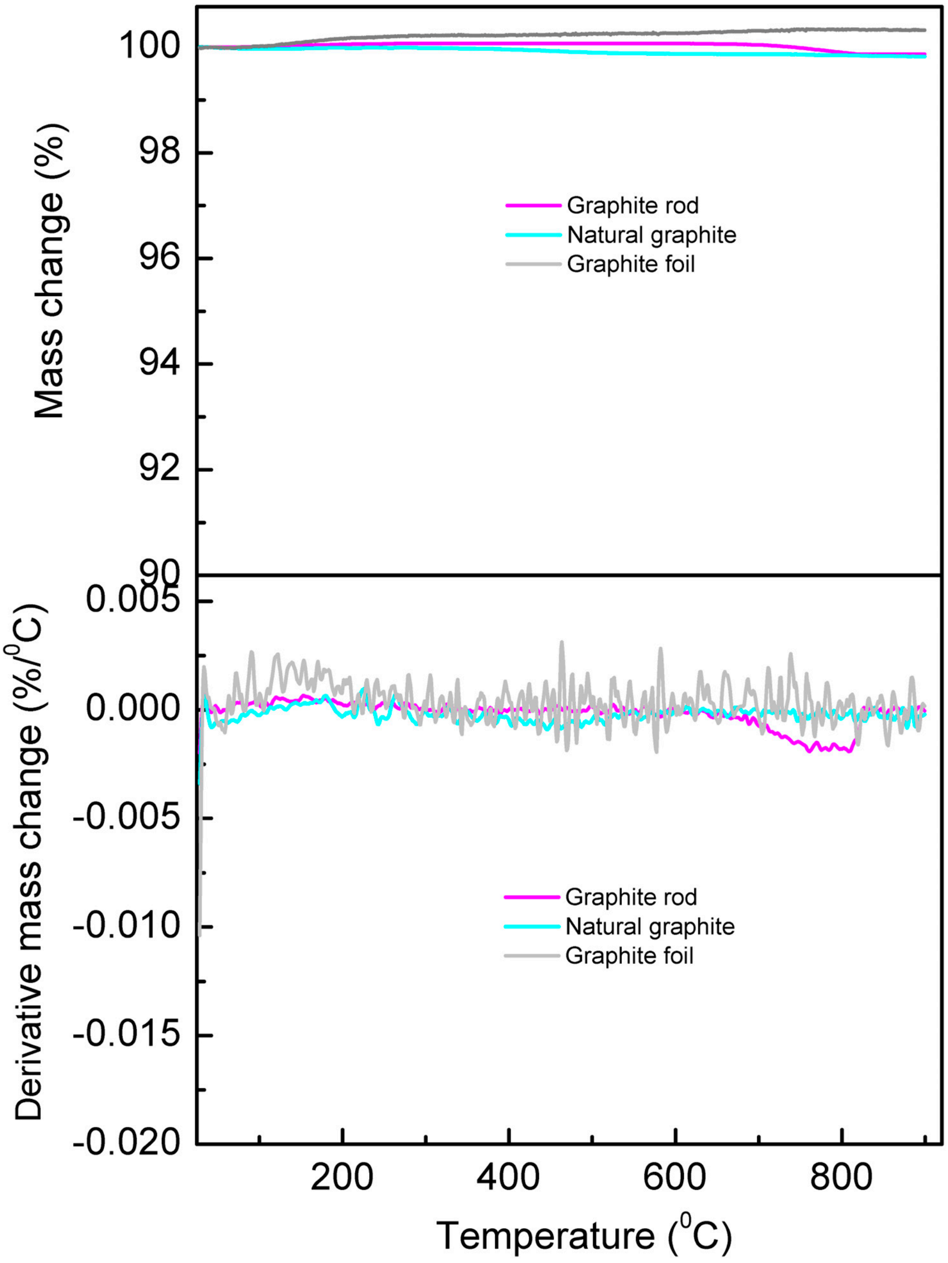

Figure S2. The TGA (top) and DTG (bottom) measurements of graphite rod, natural graphite and graphite foil from room temperature (25 ^{0}C) to 900 °C under N_2 atmosphere.

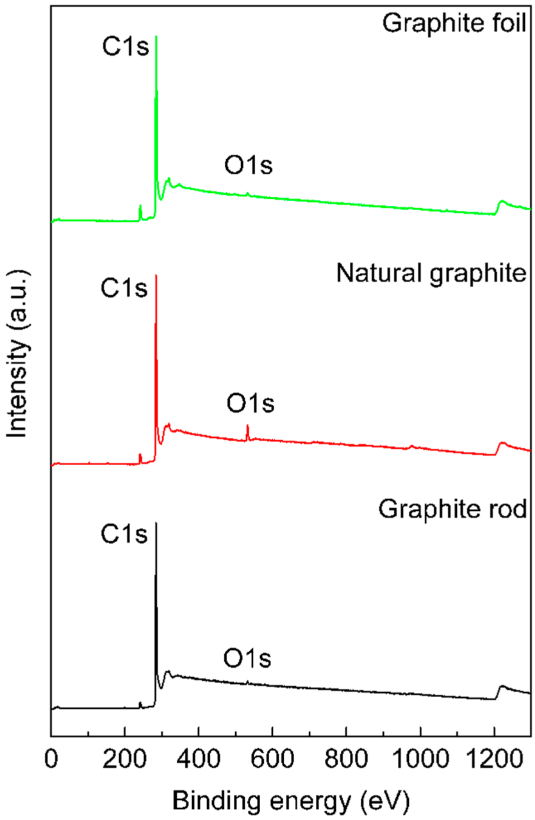

Figure S3 XPS survey scans of graphite rod, natural graphite and graphite foil, respectively.

References:

[1] Cao, J.; He, P.; Mohammed, M.A.; Zhao, X.; Young, R.J.; Derby, B.; Kinloch, I.A.; Dryfe, R.A.W. Two-Step Electrochemical Intercalation and Oxidation of Graphite for the Mass Production of Graphene Oxide. *J. Am. Chem. Soc.* **2017**, *139*, 17446.

[2] Parvez,K.; Wu, Z.-S;. Li, R.; Liu, X.; Graf, R.; Feng, X.; Müllen, K. Exfoliation of graphite into graphene in aqueous solutions of inorganic salts. *J. Am. Chem. Soc.* **2014**, *136*, 6083.

[3] Tian, S.; Yang, S.; Huang, T.; Sun, J.; Wang, H.; Pu, X.; Tian, L.; He, P.; Ding, G.; Xie, X. One-step fast electrochemical fabrication of water-dispersible graphene. *Carbon* **2017**, *111*, 617.

[4] Wang, H.S.; Tian, S.Y.; Yang, S.W.; Wang, G.; You, X.F.; Xu, L.X.; Li, Q.T.; He, P.; Ding, G.Q.; Liu, Z.; Xie, X.M. Anode coverage for enhanced electrochemical oxidation: a green and efficient strategy towards water-dispersible graphene. *Green Chem.* **2018**, *20*, 1306.

[5] Ambrosi, A.; Pumera, M. Electrochemically Exfoliated Graphene and Graphene Oxide for Energy Storage and Electrochemistry Applications. *Chem.-Eur. J.* **2016**, *22*, 153.

[6] Parvez, K.; Li, R.; Puniredd, S.R.; Hernandez, Y.; Hinkel, F.; Wang, S.; Feng, X.; Müllen, K. Electrochemically Exfoliated Graphene as Solution-Processable, Highly Conductive Electrodes for Organic Electronics. *ACS Nano,* **2013**, *7*, 3598.

[7] Xuhua, H.; Senlin, L.; Zhiqiang, Q.; Wei, Z.; Wei, Y.; Yanyan, F. Low defect concentration few-layer graphene using a two-step electrochemical exfoliation. *Nanotechnology,* **2015**, *26,* 105602.

[8] Yang, S.; Brüller, S.; Wu, Z.-S.; Liu, Z.; Parvez, K.; Dong, R.; Richard, F.; Samorì, P.; Feng, X.; Müllen, K. Organic Radical-Assisted Electrochemical Exfoliation for the Scalable Production of High-Quality Graphene. *J. Am. Chem. Soc.* **2015**, *137*, 13927-13932.

[9] Munuera, J.M.; Paredes, J.I.; Villar-Rodil, S.; Martínez-Alonso, A.; Tascón, J.M.D. A simple strategy to improve the yield of graphene nanosheets in the anodic exfoliation of graphite foil. *Carbon* **2017**, *115*, 625-628.

[10] Yu, P.; Tian, Z.; Lowe, S.E.; Song, J.; Ma, Z.; Wang, X.; Han, Z.J.; Bao, Q.; Simon, G.P.; Li, D.; Zhong, Y.L. Mechanically-Assisted Electrochemical Production of Graphene Oxide. *Chem. Mater.* **2016**, *28*, 8429-8438.

[11] Chen, C.-H.; Yang, S.-W.; Chuang, M.-C.; Woon, W.-Y.; Su, C.-Y. Towards the continuous production of high crystallinity graphene via electrochemical exfoliation with molecular in situ encapsulation. *Nanoscale*, **2015**, *7*, 15362-15373.

[12] Shi, P.C.; Guo, J.P.; Liang, X.; Cheng, S.; Zheng, H.; Wang, Y.; Chen, C.H.; Xiang, H.F. Large-scale production of high-quality graphene sheets by a non-electrified electrochemical exfoliation method. *Carbon*, **2018**, *126*, 507-513.

[13] Abdelkader, A.M.; Kinloch, I.A.; Dryfe, R.A.W. Continuous Electrochemical Exfoliation of Micrometer-Sized Graphene Using Synergistic Ion Intercalations and Organic Solvents *ACS Appl. Mater. Interfaces*, **2014**, *6*, 1632-1639.

[14] Wu, L.; Li, W.; Li, P.; Liao, S.; Qiu, S.; Chen, M.; Guo, Y.; Li, Q.; Zhu, C.; Liu, L. Powder, Paper and Foam of Few-Layer Graphene Prepared in High Yield by Electrochemical Intercalation Exfoliation of Expanded Graphite. *Small*, **2014**, *10*, 1421-1429.

[15] Zhang, Y.; Xu, Y.; Liu, R. Regulating cations and solvents of the electrolyte for ultra-efficient electrochemical production of high-quality graphene. *Carbon* **2021**, *176,* 157-167.

[16] Zhang, Y.; Xu, Y.; Zhu, J.; Li, L;. Du, X.; Sun, X. Electrochemically exfoliated high-yield graphene in ambient temperature molten salts and its application for flexible solid-state supercapacitors, *Carbon.* **2018**, *127,* 392-403.

[17] Wang, G.; Wang, B.; Park, J.; Wang, Y.; Sun, B.; Yao, J. Highly efficient and large-scale synthesis of graphene by electrolytic exfoliation. *Carbon*. **2009**, *47*, 3242-3246.

[18] Rao, K.S.; Sentilnathan, J.; Cho, H.-W.; Wu, J.-J.; Yoshimura, M. Soft Processing of Graphene Nanosheets by Glycine-Bisulfate Ionic-Complex-Assisted Electrochemical Exfoliation of Graphite for Reduction Catalysis. *Adv. Funct. Mater.* **2015**, *25*, 298-305.

[19] Cooper, A.J.; Wilson, N.R.; Kinloch, I.A.; Dryfe, R.A.W. Single stage electrochemical exfoliation method for the production of few-layer graphene via intercalation of tetraalkylammonium cations. *Carbon*, **2014**, *66*, 340-350.

[20] Kamali, A.R.; Fray, D.J. Large-scale preparation of graphene by high temperature insertion of hydrogen into graphite. *Nanoscale,* **2015**, *7*, 11310-11320.

[21] Zhao, M.; Guo, X.Y.; Ambacher, O.; Nebel, C.E.; Hoffmann, R. Electrochemical generation of hydrogenated graphene flakes. *Carbon*, **2015**, *83*, 128-135.

[22] Yang, Y.; Lu, F.; Zhou, Z.; Song, W.; Chen, Q.; Ji, X. Electrochemically cathodic exfoliation of graphene sheets in room temperature ionic liquids N-butyl, methylpyrrolidinium bis(trifluoromethylsulfonyl)imide and their electrochemical properties. *Electrochim. Acta* **2013**, *113*, 9-16.

[23] Wang, J.; Manga, K.K.; Bao, Q.; Loh, K.P. High-Yield Synthesis of Few-Layer Graphene Flakes through Electrochemical Expansion of Graphite in Propylene Carbonate Electrolyte. *J. Am. Chem. Soc.* 2011, *133*, 8888-8891.
